# Vitamin D_3_ deficiency induced intestinal inflammatory response of turbot through nuclear factor-κB/inflammasome pathway, accompanied by the mutually exclusive apoptosis and autophagy

**DOI:** 10.3389/fimmu.2022.986593

**Published:** 2022-09-08

**Authors:** Zhichu Chen, Dong Huang, Prakaiwan Yongyut, Guangbin Li, María Ángeles Esteban, Orapint Jintasataporn, Junming Deng, Wenbing Zhang, Qinghui Ai, Kangsen Mai, Yanjiao Zhang

**Affiliations:** ^1^ The Key Laboratory of Aquaculture Nutrition and Feed (Ministry of Agriculture) and the Key Laboratory of Mariculture (Ministry of Education), Ocean University of China, Qingdao, China; ^2^ Fish Innate Immune System Group, Department of Cell Biology and Histology, Faculty of Biology, University of Murcia, Murcia, Spain; ^3^ Department of Aquaculture, Faculty of Fisheries, Kasetsart University, Bangkok, Thailand; ^4^ Laboratory of Aquatic Animal Nutrition and Feed, Fisheries College, Guangdong Ocean University, Zhanjiang, China

**Keywords:** vitamin D_3_, vitamin D_3_ receptor, NF-κB, inflammasome, inflammation, apoptosis, autophagy

## Abstract

Vitamin D_3_ (VD_3_) participated widely in the nuclear factor-κB (NF-κB)-mediated inflammation, apoptosis, and autophagy through the vitamin D receptor (VDR). However, the molecular mechanisms remain not understood in teleost. The present study investigated the functions of VD_3_/VDR on intestinal inflammation, autophagy, and apoptosis of turbot *in vivo* and *in vitro*. Triple replicates of 30 fish were fed with each of three diets with graded levels of 32.0 (D_0_), 1012.6 (D_1_), and 3978.2 (D_2_) IU/kg VD_3_. Obvious intestinal enteritis was observed in the D_0_ group and followed with dysfunction of intestinal mucosal barriers. The intestinal inflammatory response induced by VD_3_ deficiency was regulated by the NF-κB/inflammasome signalling. The promotion of intestinal apoptosis and suppression of intestinal autophagy were also observed in the D_0_ group. Similarly, VD_3_ deficiency *in vitro* induced more intense inflammation regulated by NF-κB/inflammasome signalling. The mutually exclusive apoptosis and autophagy were also observed in the group without 1,25(OH)_2_D_3_ *in vitro*, accompanied by similar changes in apoptosis and autophagy increased apoptosis. The gene expression of VDRs was significantly increased with the increasing VD_3_ supplementation both *in vivo* and *in vitro*. Moreover, VDR knockdown in turbot resulted in intestinal inflammation, and this process relied on the activation of inflammasome mediated by NF-κB signalling. Simultaneously, intestinal apoptosis was promoted, whereas intestinal autophagy was inhibited. In conclusion, VD_3_ deficiency could induce intestinal inflammation *via* activation of the NF-κB/inflammasome pathway, intestinal apoptosis, and autophagy formed a mutually exclusive relation in teleost. And VDR is the critical molecule in those processes.

## 1 Introduction

In fish, the intestine is a major immune organ that provides a tight barrier against pathogenic infections and coexists with many commensal organisms while absorbing and metabolizing nutrients (1, 2). The pathological process of enteritis in fish is closely associated with dysfunction of intestinal mucosal barriers, including the overexpression of pro-inflammatory cytokines, abnormal tight junction protein assembly, and decreased mucin secretion (3–6). In a variety of nutrients that can enhance the intestinal health of aquatic animals, it has been widely proven that dietary vitamin D_3_ (VD_3_) could improve intestinal digestion and utilization of nutrients, alleviating intestinal inflammation (5, 7, 8). However, there is no existent for the autonomous synthesis of VD_3_ in fish (9). Generally, fish need vitamin D_3_ supplements through feed and then metabolize it into 1,25(OH)_2_D_3_ and exert biological activity.

In mammals, VD_3_ signalling through its nuclear vitamin D receptor (VDR) has emerged as a key immune system modulator. Decreased serum 1,25(OH)_2_D_3_ levels or VD_3_ deficiency has been linked to human intestinal diseases, such as inflammatory bowel diseases and short bowel syndrome (10–12). In the development of intestinal inflammation, there was an important role of VD_3_ and VDR in maintaining intestinal barrier function and innate antibacterial immunity. Such as, in Caco-2 cells, 1,25(OH)_2_D_3_ increased junction protein expression and intestinal transepithelial electric resistance and preserved the integrity of tight junctions in the presence of dextran sulfate sodium (DSS) (13). Administration of 1,25(OH)_2_D_3_ downregulates cadherin-17 but upregulates tight junction proteins (claudin 2 and 12) expression in the intestine of calbindin-D_9k_ null mutant mice (14). Furthermore, VDR deletion in intestinal epithelial cells leads to the decreased expression of claudin 2 and 12 in the intestine of mice and intestinal cell line Caco-2 (15). In addition, the activation of the NF-κB pathway by VD_3_ was also observed in juvenile Chinese mitten crabs (*Eriocheir sinensis*) that VD_3_ might improve intestinal immunity *via* the VDR/TLR/MyD88/NF-κB pathway (5). However, whether VD_3_/VDR mediated intestinal barrier function and NF−κB signalling pathway involved in regulating intestinal inflammation is not comprehensively understood in the teleost.

Excess cytokine production by activated intraepithelial lymphocytes and other immune cells can induce apoptosis directly by suppressing anti-apoptotic signals in the epithelium (16–18). Additionally, autophagy plays a key role in the prevention of intestinal inflammation, impaired autophagy exhibit exacerbated colitis induced by DSS in different autophagy-deficient mice models (19–22), and the activation of autophagy suppressed intestinal inflammation in experimental models of colitis and Crohn’s disease (23–25). Moreover, VD_3_ has been regarded as an opponent of colorectal cancer by inhibiting epithelial cell apoptosis, while an increased number of apoptotic cells was observed in the small intestine of VDR-deficient mice (26–28). Furthermore, apoptosis and autophagy balance is critical for maintaining the normal functions of the intestine (27). Previous studies have shown that the regulation of intestinal epithelial VDR depends on the autophagy pathway of autophagy related 16 like protein 1 (ATG16L1), while ATG16L1 was regulated by activated caspase-3 in the process of apoptosis (29–31). A similar result in abalone showed that dietary VD_3_ can inhibit apoptosis and produce autophagy simultaneously (32). However, the regulation of VD_3_/VDR in the interaction of intestinal apoptosis and autophagy in teleost is still unknown.

Turbot (*Scophthalmus maximus* L.) is cultured wildly in the world. In recent years, more and more attention has been paid to regulating intestinal health through nutritional strategy in the healthy cultivation of fish. Therefore, the present study aimed to evaluate the role of VD_3_ in regulating intestinal inflammation and the relationship between intestinal apoptosis and autophagy in turbot.

## 2 Materials and methods

### 2.1 Experiment 1: Diets, fish husbandry, and sampling of VD_3_ treatments *in vivo*

As shown in **Table 1**, three isonitrogenous and isolipidic experimental diets were formulated to contain approximately 50% crude protein and 10% crude lipid. The basal diet used casein (vitamin free), gelatine, and crystalline amino acid as the primary protein sources, while fish oil, soya bean oil, and soya bean lecithin were used as dietary lipid sources. VD_3_ (V8070, Solarbio, China) was added to the basal diet to provide graded concentrations of 0 (D_0_), 1000 (D_1_), and 4000 (D_2_) IU/kg VD_3_. The measured values of VD_3_ concentration were determined by the high-performance liquid chromatography (HPLC) method as described previously (33), which were 32 (D_0_), 1012.60 (D_1_), and 3978.2 (D_2_) IU/kg. Diets were extruded with an experimental single-screw feed mill (Yihe, China) in the form of 3 mm diameter pellets and dried for 12 h in a ventilated oven at 50°C. All the experimental diets were stored at −20°C until use.

**Table 1:** Formulation and proximate composition of the experimental diets (% dry matter).

Ingredients	Diets		
	D0	D1	D2
Casein (vitamin free)	37.20	37.20	37.20
Gelatin	9.30	9.30	9.30
Crystalline amino acid ^a^	5.60	5.60	5.60
Fish oil	5.50	5.50	5.50
Soya oil	4.10	4.10	4.10
Soybean lecithin	1.00	1.00	1.00
Choline chloride	0.30	0.30	0.30
Ca(H_2_PO_4_)_2_·H_2_O	1.50	1.50	1.50
Ethoxyquin	0.05	0.05	0.05
Y_2_O_3_	0.10	0.10	0.10
Calcium propionate	0.10	0.10	0.10
Phagostimulant ^b^	1.00	1.00	1.00
Dextrin	26.50	26.50	26.50
Microcrystalline cellulose	5.75	5.75	5.75
Mineral premix ^c^	1.00	1.00	1.00
Vitamin premix ^d^	1.00	1.00	1.00
Vitamin D_3_ (add value, IU/kg)	0.00	1,000.00	4,000.00
***Proximate composition***			
Vitamin D_3_ (measured value, IU/kg)	32.00	1,012.60	3,978.20
Moisture	9.83	9.21	10.09
Crude protein	50.29	50.14	50.11
Crude lipid	9.39	9.59	9.65
Ash	3.44	3.40	3.42

a Crystalline amino acid premix (g/100 g diet): arginine, 1.69; histidine, 0.55; isoleucine, 0.22; leucine, 0.14; lysine, 0.73; phenylalanine, 0.50; threonine, 0.61; valine, 0.13; alanine, 1.32; aspartic acid, 1.63; glycine, 1.62; serine, 0.42; cystine, 0.40; tyrosine, 0.10.

b Phagostimulant (g/kg diet): betaine, 4; DMPT, 2; threonine, 2; glycine, 1; inosine-5′-diphosphate trisodium salt, 1.

c Mineral premix (mg/kg diet): FeSO_4_·H_2_O, 80; ZnSO_4_·H_2_O, 50; CuSO_4_·5H_2_O, 10; MnSO_4_·H_2_O, 45; KI, 60; CoCl_2_·6H_2_O (1%), 50; Na_2_SeO_3_ (1%), 20; MgSO_4_·7H2O, 1200; zeolite, 8485.

d Vitamin premix (mg/kg diet): retinyl acetate, 32; DL-α-tocopherol acetate, 240; vitamin K_3_, 10; thiamin, 25; riboflavin (80%), 45; pyridoxine hydrochloride, 20; vitamin B_12_ (1%), 10; Lascorbyl-2-monophosphate-Na (35%), 2000; calcium pantothenate, 60; nicotinic acid, 200; inositol, 800; biotin (2%), 60; folic acid, 20; cellulose, 6478.

Disease-free juvenile turbots were purchased from a local farm in Weihai, Shandong Province, China. Before the start of the feeding trial, the fish were maintained for two weeks to acclimate to the experimental conditions. A commercial diet (Surgreen, China) was fed during the acclimation, which is specially formulated for the nutritional requirements of the turbot. The fish were then fasted for 24 h and weighed. A total of 270 fish (12.17 g initial body weight) were randomly assigned to 9 fiberglass tanks (300 L, 30 fish per tank) connected to an indoor flow-through water system. Triplicate tanks of fish were fed with each experimental feed to apparent satiation twice daily for 12 weeks. During the feeding period, water temperature ranged from 15°C to 18°C; salinity 30-33‰; and dissolved oxygen higher than 7.0 mg/L.

Three fish were selected randomly from each tank for the sampling. All fish were anesthetized with eugenol (1:10000, Shanghai Reagent Corp, China) before handling. The intestinal tissue samples for the analysis of enzymes activities, DNA laddering, gene expression, and western blot were frozen in liquid nitrogen, and the others for the analysis of histology were fixed in Bouin’s fixative solution.

### 2.2 Experiment 2: Cell culture and sampling of VD_3_ treatments *in vitro*

According to our previous study (34), the isolation and culture of primary intestinal epithelial cells were performed. Briefly, the starved turbots were anesthetized with eugenol (1:10000, Shanghai Reagent Corp, China), wiped, and disinfected with 75% ethanol. Then the intestine tissue was dissected and rinsed repeatedly with the solution of Hank’s Balanced Salt Solution (HBSS) containing penicillin-streptomycin (Thermo Fisher Scientific, USA). About 1 mm^3^ of intestinal tissues were dissociated with collagenase and dispase (Thermo Fisher Scientific, USA) for 15 to 20 min. The enzyme solution was washed several times with L-15 medium (L5520, Sigma, USA), and the supernatants were collected and centrifuged for 5 min at 1000 rpm to obtain intestinal epithelial cells suspended in the supernatants.

The intestinal epithelial cells were seeded into 6-well plates at a density of 1.0×10^6^ cells/mL with a modified L-15 medium (L5520, Sigma, USA) containing 5% fetal bovine serum (FBS, Biological Industries, Israel), 10 ng/mL epidermal growth factor (Sigma, USA), 0.2% insulin transferrin-selenium-sodium pyruvate (Thermo Fisher Scientific, USA), antifungal, and antibacterial substances at 23°C in an incubator (CO_2_-free). After the cells adhered to the well for 24 h, the cells were treated with fresh medium together with 0, 1, 10, and 50 nM 1,25(OH)_2_D_3_ (HY-10002, Med Chem Express, USA). After 1,25(OH)_2_D_3_ treatments for 24 h, the cell lysates were subjected to RNA and protein extraction, while the cell climbing slides were fixed with 4% paraformaldehyde for TUNEL assay and MAP1LC3B immunofluorescence.

### 2.3 Experiment 3: RNA interference of VDRs *in vivo* and sampling

Three pairs of VDR-specific siRNAs and scrambled siRNA for negative control (NC siRNA) set up through BLOCK-iT™ RNAi Express are shown in **Table 2** and **Figure S**, and synthesized by T7 RNAi Transcription Kit (TR102, Vazyme, China) according to the instructions. Seven groups of 9 turbots acclimating to an indoor flow-through water system were injected with VDR-specific siRNAs (1 μg/g) and NC siRNA (1 μg/g), and the intestinal tissues were drawn to analyze the gene expression of VDRA&B gene expression after 24 h. Three turbots were mixed together to make a sample. And the most effective siRNA of VDRA&B was selected for the formal experiment.

**Table 2:** siRNA sequences.

	Sequence
VDRA siRNA 1	CAATGTTTCTAGATGTTTA
VDRA siRNA 2	GTTCAAGATTGTAAATCAA
VDRA siRNA 3	GCTGTGGTGTAGCAAGTTA
VDRB siRNA 1	GGACCTTCATATAATACAA
VDRB siRNA 2	GGTTGATGTATTTGACTAA
VDRB siRNA 3	GGATGTACACCATACTCTA
NC siRNA	GCTGACCCTGAAGTTCATC

The formal *RNA interference* (RNAi) experiment lasted for two days. The body weight of the fish decided the dose of siRNA (1 μg/g). Five groups of 9 turbots were injected with equal volume (100 μl) of PBS, PBS, NC siRNA, VDRA siRNA, and VDRB siRNA on the first day, respectively. After 24 h, they continued to be injected with the same contents as the first day. In addition to the first group still injected with PBS, the last four groups were injected with lipopolysaccharides (LPS) at the rate of 2.5 μg/g body weight. They were defined as PBS, LPS, NC siRNA+LPS, VDRA siRNA+LPS, and VDRB siRNA+LPS group. The turbots were anesthetized by eugenol (1:10000, Shanghai Reagent Corp, China) and had their intestinal tissue taken for qRT-PCR and western blot.

### 2.4 Chemical analysis of diets

Standard methods (AOAC, 1995) were used for analyzing experimental diets. Moisture content was determined gravimetrically to constant weight in an oven at 105°C. Crude protein was determined by the Kjeldahl method using Kjeltec 2300 (Foss, Denmark) using boric acid to trap released ammonia. Crude lipid was determined by petroleum ether extraction using Soxhlet Extraction System B-811 (Buchi, Switzerland). Ash was determined by combustion at 550°C.

### 2.5 Intestinal histology

After being fixed in Bouin’s fixative solution for 24 h, the intestinal samples were transferred to 70% ethanol and embedded in paraffin after dehydration. Paraffin sections of 5 μm were cut and stained with hematoxylin and eosin (H&E, G1120, Solarbio, China) according to the manufacturer’s protocol. The slides were examined under a light microscope (BX43F, Olympus, Japan) equipped with a digital microscope camera (DP72, Olympus, Japan) for image acquisition.

### 2.6 Intestine-related enzyme activities

For the analysis of intestinal alkaline phosphatase (ALP), acid phosphatase (ACP), and lysozyme (LYZ) activities, the intestinal samples were homogenized in ice-cold physiological saline solution (1:9) and centrifuged at 2500 g for 10 min at 4°C. The relevant intestinal enzyme activities were then determined with supernatants, whose protein concentrations were measured by the bicinchoninic acid (BCA) protein analysis kit (P0012, Beyotime, China). The activities of ALP (A059-2), ACP (A060-2), and LYZ (A050-1) were quantified using the commercial kits according to the manufacturer’s protocol (Jiancheng Bioengineering Institute, China).

### 2.7 DNA fragmentation assay

Briefly, 5 mg intestinal tissues were homogenized in liquid nitrogen and then mixed with 0.5 ml DNA extraction lysis buffer (pH 8.0) containing 50 mM Tris-HCl (Solarbio, China), 25 mM EDTA (Solarbio, China), 100 mM NaCl (Macklin, China), 1% Triton X-100 (Solarbio, China), and 0.5 mg/ml Protease K (Solarbio, China). The lysates were incubated overnight at 50°C with gentle shaking and then mixed with an equal volume of phenol-chloroform-isoamyl alcohol (25:24:1) (Solarbio, China). After being precipitated with 5 M ammonium acetate (Solarbio, China) and absolute ethanol (Sangon, China), the DNA precipitations were washed three times with 0.6 ml 70% ethanol and air-dried at room temperature (RT). The DNA precipitations were dissolved with Tris-EDTA buffer (Solarbio, China) containing 100 µg/ml RNase A (Solarbio, China) and incubated at 30°C for 30 min. Approximately 5 µg of DNA samples were electrophoresed (100 V) on a 2% agarose gel and were visualized with Gel-Red (TransGen Biotech, China) and recorded under UV light with an Odyssey Infrared Imaging System (Li-Cor Bioscience, USA). Trans2K^®^ Plus DNA Marker (BM111, TransGen, China) of nuclei acid was used as the reference. The evaluation of DNA fragmentation was determined according to Yuan et al. (35). The density of the 180 to 200 bp DNA band was quantified using ImageJ software (National Institutes of Health, USA), and the relative density was normalized to the D_0_ group.

### 2.8 Tunel, immunofluorescence, and photomicrograph

TMR (red) Tunel Cell Apoptosis Detection Kit (G1502, Servicebio, China) was used to detect positive apoptotic nuclei of cell climbing slides. The cell climbing slides were dried and then incubated in the permeabilizing work solution for 20 min at RT. Rinse with PBS solution three times, each for 5 min. After the slices were slightly dried, the equilibration buffer was added to the tissues and incubated for 10 min at RT. After washing with PBS solution three times (5 min per time), the TUNEL reaction solution mixture (TDT enzyme, dUTP, and buffer at 1:5:50 ratio) was added to objective tissue placed in a flat wet box, incubated for 2 h at 37°C. 4’,6-diamidino-2-phenylindole (DAPI) was used to stain the nucleus and washed out three times with PBS in a rocker device.

Immunofluorescence was used for detecting MAP1LC3B in climbing slides. The cell membrane rupture was conducted as mentioned above, and then the slides were incubated in 5% Bovine Serum Albumin (BSA) for 30 min at RT. MAP1LC3B antibody (AF5225, Beyotime, China) was added to the sections, and the slides were incubated overnight at 4°C. After being washed with PBS solution three times (5 min per time), the tissues were covered with the secondary antibody and incubated for 50 min at RT. The nucleus was stained by DAPI and washed three times with PBS.

The sections were observed under a fluorescence microscope, and images were collected. (DAPI UV excitation wavelength 330-380 nm, emission wavelength 420 nm, blue light emission; CY3 excitation wavelength 510-561 nm, emission wavelength 590 nm, red light emission).

### 2.9 RNA extraction and quantitative real-time PCR (qRT-PCR)

RNA was extracted from the intestinal samples with Trizol Reagent (Takara, Japan). The integrity of RNA was detected by electrophoresis on 1% denaturing agarose gel, and the concentration was detected with a Nano Drop^®^2000 spectrophotometer (Thermo Fisher Scientific, USA). A total of 1 μg RNA was reversely transcribed to cDNA with Evo M-MLV Mix Kit with gDNA Clean for qPCR [#AG11728, Accurate Biotechnology (Hunan) Co., Ltd., China].

The gene expression of VD_3_ receptor A and B (*VDRA&B*), interleukin 1 beta (*IL1B*), interleukin 8 (*IL8*), tumor necrosis factor (*TNF*), and interferon gamma (*IFNG*), apoptosis-associated speck-like protein containing a caspase recruitment domain (*ASC*), transforming growth factor beta 1 (*TGFB1*), and interleukin-10 (*IL 10*), B cell lymphoma 2 (*BCL2*), caspase 3 (*CASP3*), microtubule-associated protein 1 light chain 3 beta (*MAP1LC3B*), and autophagy-related 16 like 1 (*ATG16L1*) was tested in the present study. Glyceraldehyde-3-phosphate dehydrogenase (*GAPDH*) and (*RPSD*) RNA polymerase II subunit D were used as the housekeeping genes. Specific primers for target genes and housekeeping genes (**Table 3**) were synthesized by Sangon (China), and the application efficiency was then assessed. Quantitative PCR was conducted in an ABIPRISM 7500 Instrument (Applied Biosystems, USA) with ChamQ Universal SYBR qPCR Master Mix (#Q711, Vazyme, China). The relative gene expression of genes was calculated using the 2^–ΔΔCT^ method (36).

**Table 3:** Primers used in quantitative real-time PCR (qRT-PCR).

Target gene	Forward primer (5′-3′)	Reverse primer (5′-3′)	GenBank no.
*MUC2*	ATGTGGAGTGTGTCGGCTT	AGACCTTGCACTGCATCTG	MF370857.1
*MUC18*	TTGTCCCTGACCAAGTGATG	ACAAAGCCTGTCCAAGATCG	JU370277.1
*CLDN4*	ATGTGGAGTGTGTCGGCTT	AGACCTTGCACTGCATCTG	MF370857.1
*TRIC*	GCCTACATCCACAAAGACAACG	TCATTCCCAGCACTAATACAATCAC	KU238183.1
*JAM1*	CCAAGATGGACACCGGAACT	CCTCCGGTGTTTAGGTCACG	MT787206.1
*ZO1*	GAGTTTTCAGCTTCCGTGTT	AGAGAACCTGTCACTGATAGATGC	KU238184.1
*VDRA*	CCACTTCAATGCCATGACC	TACTGCGCCTAAAGAACCCT	XM_035644868.1
*VDRB*	TAATGGCAGTTGCACCATCACC	TCCTCTGCACTTCCTCGTCT	XM_035630011.1
*IL1B*	ATGGTGCGATTTCTGTTC	CACTTTGGGTCGTCTTTG	AJ295836.2
*TNF*	GGACAGGGCTGGTACAACAC	TTCAATTAGTGCCACGACAAAGAG	AJ276709.1
*IL8*	GGAATTAATCCCTGGCAACTCT	ACCTCTTTGCCTGAGTGT	XM_035638412.1
*IFNG*	GCTTTCCCGATCATCTTCTG	GGTTTCCCAGATTCCCATTC	DQ400686.1
*TGFB1*	CTGCAGGACTGGCTCAAAGG	CATGGTCAGGATGTATGGTGGT	KU238187.1
*IL10*	TCGACGAGCTCAAGTCCGAT	CTGATCCAGCTCGCCCAT	XM_035632547.1
*CASP3*	TCGTTCGTCTGTGTCCTGTTGAG	GCTGTGGAGAAGGCGTAGAGG	XM_035637276.1
*BCL2*	GTGAACTGGGGCCGGATTATC	CCATCCCCCGTTGTCCATAAT	XM_035631516.1
*BAX*	GCTCCAGAGGATGATAAATAAC	AAAGTAGAAGAGTGCGACCA	XM_020094597.1
*MAP1LC3B*	GCACCCCAACAAGATCCCT	GATCTTGACCAGCTCGCTCA	XM_035642079.1
*ATG16L1*	GCAGATCACATTCTCCGCTCT	TGCCATCCAGCGAGACACC	XM_035621910.1
*GAPDH*	CAGTGTATGAAGCCAGCAGAG	GGTCGTATTTGTCCTCATTAACTC	AY008305.1
*RPSD*	AACACAGGAAGCAGCAGAAC	ACGGCAGTGATGGTCTCTC	DQ848899.1

### 2.10 Western blot

The intestinal tissues were dissolved with RIPA lysis buffer (Solarbio, China) with the protease and phosphatase inhibitor (Roche, Switzerland). After centrifuging at 12000 g for 20 min at 4°C, the supernatants of the lysates were collected as the total proteins. The nuclear proteins of intestinal tissue were extracted using NE-PER™ Nuclear and Cytoplasmic Extraction Reagents (Thermo Fisher Scientific, USA), as the manufacturer’s protocol showed. And then, the protein concentrations of the samples mentioned above were measured by the bicinchoninic acid (BCA) protein analysis kit (P0012, Beyotime, China). The standardized samples were mixed with Omni-Easy™ Protein Sample Loading Buffer (EpiZyme, China). A total of 20 µg of protein was loaded and separated by sodium dodecyl sulfate-polyacrylamide gel electrophoresis. The proteins in the gel were transferred to a polyvinylidene difluoride (PVDF) membrane (Millipore, USA) for 1 h at 70 V, followed by membrane blocking at RT for 2 h with 5% non-fat milk (Sangon, China) in tris-buffered saline with Tween 20 (TBST). The membrane was incubated with primary antibodies overnight at 4°C and then washed three times for 5 min each with TBST. Next, the membrane was incubated with horseradish peroxide (HRP)-conjugated secondary antibody (A0208, Beyotime, China) dissolved with 1% non-fat milk in the TBST for 1 h at RT. After washing with TBST 3 times for 5 min, the membrane was developed with enhanced chemiluminescence (Vazyme, China) according to the manufacturer’s directions. The blots were recorded with an Odyssey Infrared Imaging System (Li-Cor Bioscience, USA). The following antibodies were used: antibodies against ASC (WL02462, Wanleibio, China), NF-κB p65 (8242, CST, USA), phos-IκBα ^ser32 ser36^ (2859, CST, USA), IκBα (WL01936, Wanleibio, China), Cleaved-caspase 3 (WL02117, Wanleibio, China), BCL2 (WL01556, Wanleibio, China), MAP1LC3B (AF5225, Beyotime, China), ATG16L1 (AF6252, Beyotime, China), Lamin B (AF1408, Beyotime, China), GAPDH (AB-P-R001, GoodHere, China). All the band intensities were quantified using ImageJ software (National Institutes of Health, USA). Respectively, the densities of total protein bands and nuclear proteins were normalized to GAPDH or Lamin B, which served as internal controls.

### 2.11 Statistical analysis

Data were analyzed by one-way analysis of variance (ANOVA) using IBM SPSS Statistics for WINDOWS Version 22.0. Tukey’s test was used to compare the means among individual treatments. Differences were regarded as significant when *P* < 0.05, and the results are presented as means ± standard deviation (SD).

## 3 Results

### 3.1 VD_3_ treatments *in vivo*

#### 3.1.1 Intestinal histology

**Figure 1:**
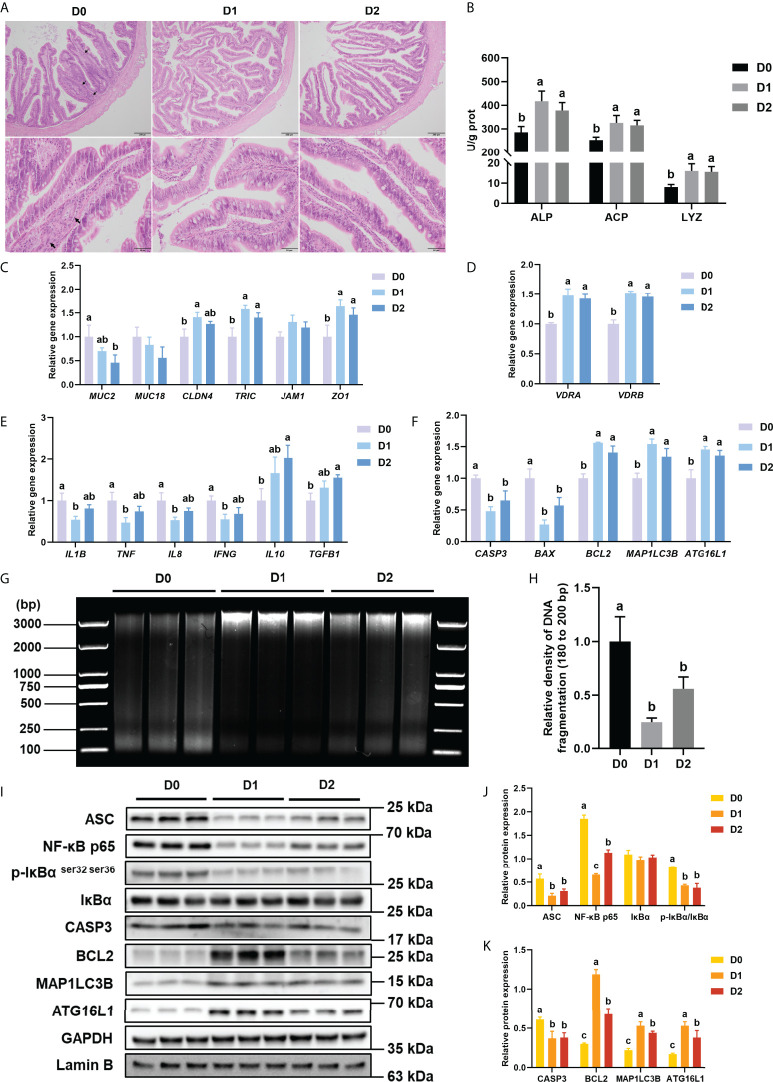
Inflammation, apoptosis, and autophagy of intestine with VD_3_ treatments *in vivo*. **(A)** H&E staining of intestine (Black bars, 200 μm or 50 μm), Black arrows indicate widening of the intestinal lamina propria. **(B)** Alkaline phosphatase, acid phosphatase activities, and lysozyme activities. **(C)** The relative mRNA expression level of *MUC2*, *MUC18*, *CLDN4*, *TRIC*, *JAM1*, and *ZO1*. **(D)** The relative mRNA expression level of *VDRA&B*. **(E)** The mRNA expression levels of *IL1B*, *TNF*, *IL8*, *IFNG*, *TGFB1*, and *IL10*. **(F)** The mRNA expression levels of *CASP3*, *BCL2*, *BAX*, *MAP1LC3B*, and *ATG16L1*. **(G, H)** DNA fragmentation and quantification of the density of the 180 to 200 bp DNA band. **(I-K)** The level of ASC, intranuclear NF-κB p65, total IκB and phosphorylated IκB, CASP3, BCL2, MAP1LC3B, and ATG16L1 were analyzed and quantitated by western blot. The blots of ASC, IκBα, p-IκBα ^ser32 ser36^, CASP3, BCL2, MAP1LC3B, and ATG16L1 were used for GAPDH loading control, while the blot of NF-κB p65 was used for Lamin B loading control. Error bars of columns denote SD (n = 3), and columns with different letters above them are significantly different (*P* < 0.05).

H&E staining was used in the present study to study the effect of different VD3 levels on the intestinal histology of turbot. The represented histological sections of the distal intestine are shown in **Figure 1A**. In the D_0_ group, shortened mucosal fold, disordered goblet cells, and widened lamina propria were observed. However, the intestine of the D_1_ and D_2_ groups showed lengthened mucosal fold, well disturbing goblet cells, and reduced thickness of the lamina propria.

#### 3.1.2 Intestine-related enzyme activities *in vivo*

The activities of ALP, ACP, and LYZ were tested in the present study to reflect the intestinal immune status of turbot in different groups. Compared with the D_0_ group, the D_1_ and D_2_ diets could significantly enhance the activities of ALP, ACP, and LYZ in the intestine of turbot (*P* < 0.05) (**Figure 1B**).

#### 3.1.3 Mucins and tight junction proteins *in vivo*

Four intestinal tight junction proteins (*CLD4*, *TRIC*, *JAM1*, and *ZO1*) and two Mucins (*MUC2* and *MUC18*) gene expressions were analyzed in the present study. Compared with the D_0_ diet, the D_1_ diet significantly elevated the gene expression of *CLD4*, *TRIC*, and *ZO1* in the intestine (*P* < 0.05), whereas the D_2_ Diet prominently increased *TRIC* and *ZO1* mRNA levels (*P* < 0.05) (**Figure 1C**). Compared with the D_0_ diet, the D_2_ diet significantly decreased the gene expression of *MUC2* in the intestine (*P* < 0.05) (**Figure 1C**).

#### 3.1.4 VDRs and inflammation-related parameters *in vivo*

The gene expression of VDRs (*VDRA*&*B*), pro-inflammatory cytokines (*L1B*, *IL8*, *TNF*, and *IFNG*), and anti-inflammatory cytokines (*TGFB1* and *IL10*) was tested in the present study to indicate the intestinal inflammatory status. Compared with the D_0_ diet, the D_1_ diet significantly (*P* < 0.05) suppressed the expression of pro-inflammatory cytokines (*IL1B*, *IL8*, *TNF*, and *IFNG*) but significantly (*P* < 0.05) increased the expression of VDRs (*VDRA* and *VDRB*); D_2_ diet markedly increased the expression of *VDRA* and *VDRB*, as well as the anti-inflammatory cytokines (*TGFB1* and *IL10*) (*P* < 0.05) (**Figures 1D, E**).

And the protein level of NF-κB signaling pathway-related molecules (ASC, the ratio of phos-IκBα ^ser32 ser36^/IκBα and intranuclear NF-κB p65) was tested in this study. Total ASC, the ratio of phos-IκBα ^ser32 ser36^/IκBα and intranuclear NF-κB p65 in the intestine were significantly down-regulated by dietary VD_3_ (*P* < 0.05) (**Figure 1J**).

#### 3.1.5 Apoptosis-related parameters *in vivo*

DNA fragmentation could reflect the apoptosis level of intestine tissues. The DNA laddering of the three groups is shown in **Figure 1G**. The relative density of 180 to 200 bp DNA band was significantly (*P* < 0.05) lower in the D_1_ and D_2_ groups compared with that in the D_0_ groups (**Figure 1H**).

The gene expression of apoptosis-related genes (*CASP3*, *BAX*, and *BCL2*) was analyzed in the present study. The results of apoptosis-related gene expression showed that D_1_ and D_2_ diets significantly (*P* < 0.05) reduced the gene expression of *CASP3* and *BAX* but remarkably (*P* < 0.05) raised the gene expression of *BCL2* (**Figure 1F**).

The protein expression of apoptosis-related proteins (CASP3 and BCL2) was also analyzed. The western blot results showed that both the D_1_ and D_2_ diets significantly (*P* < 0.05) suppressed the expression of CASP3 but increased the expression of BCL2 considerably (*P* < 0.05) (**Figure 1I, K**).

#### 3.1.6 Autophagy-related parameters *in vivo*

The gene and protein expression of autophagy-related genes (ATG16L1 and MAP1LC3B) were analyzed in the present study. Dietary VD_3_ significantly (*P* < 0.05) elevated both the protein and mRNA expression of *ATG16L1* and *MAP1LC3B* (**Figures 1F, I, K**).

### 3.2 VD_3_ treatments *in vitro*

#### 3.2.1 Tight junctions *in vitro*

**Figure 2:**
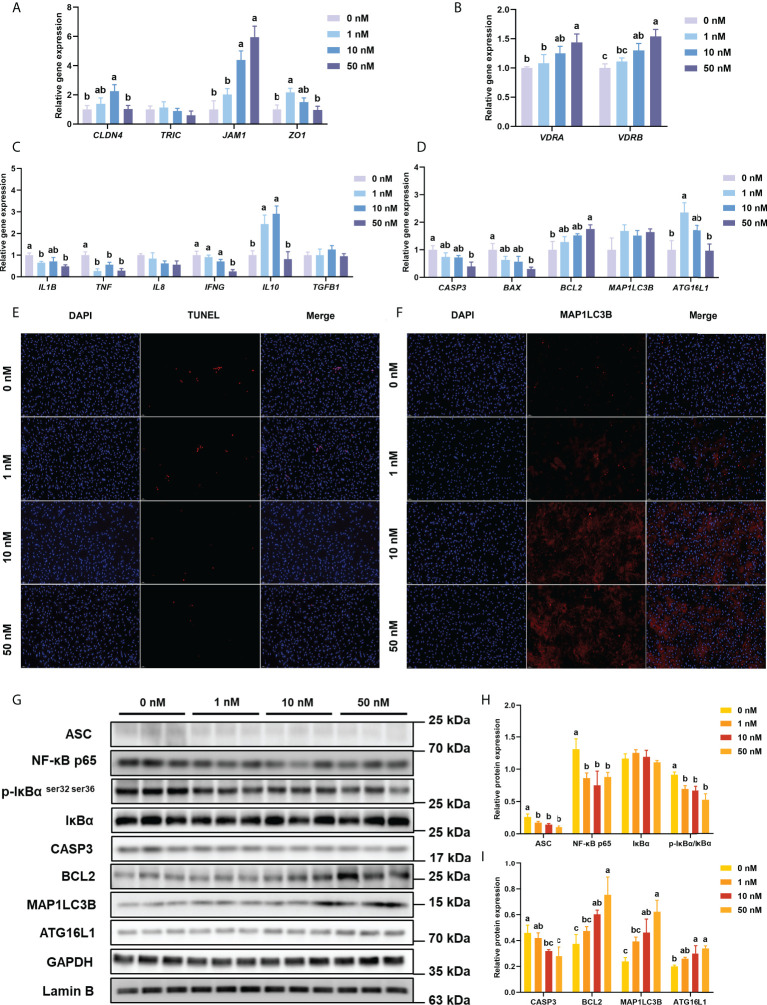
Inflammation, apoptosis, and autophagy of intestinal epithelial cells with VD_3_ treatments *in vitro*. **(A)** The relative mRNA expression level of *CLDN4*, *TRIC*, *JAM1*, and *ZO1*. **(B)** The relative mRNA expression level of *VDRA&B*. **(C)** The mRNA expression levels of *IL1B*, *TNF*, *IL8*, *IFNG*, *TGFB1*, and *IL10*. **(D)** The mRNA expression levels of *CASP3*, *BCL2*, *BAX*, *MAP1LC3B*, and *ATG16L1*. **(E)** Confocal images of TUNEL assay (White bars, 50 μm). **(F)** Confocal images of MAP1LC3B fluorescence (White bars, 50 μm). **(G-I)** The level of ASC, intranuclear NF-κB p65, total IκB and phosphorylated IκB, CASP3, BCL2, MAP1LC3B, and ATG16L1 were analyzed and quantitated by western blot. The blots of ASC, IκBα, p-IκBα ^ser32 ser36^, CASP3, BCL2, MAP1LC3B, and ATG16L1 were used for GAPDH loading control, while the blot of NF-κB p65 was used for Lamin B loading control. Error bars of columns denote SD (n = 3), and columns with different letters above them are significantly different (*P* < 0.05).

Compared with the 0 nM 1,25(OH)_2_D_3_ supplementation in intestinal epithelial cell *in vitro*, 1 nM 1,25(OH)_2_D_3_ significantly (*P* < 0.05) increased the gene expression of *ZO1*, 10 nM greatly (*P* < 0.05) increased the expression of *CLD4* and *JAM1*, and 50nM remarkably (*P* < 0.05) increased the expression of *JAM1*(**Figure 2A**).

#### 3.2.2 VDRs and inflammation-related parameters *in vitro*

With the increase of 1,25(OH)_2_D_3_ concentration in the culture medium, the expression of *VDRA&B* increased significantly (*P* < 0.05) and reached the peak at the concentration of 50 nM 1,25(OH)_2_D_3_ (**Figure 2B**). Compared with the 0 nM group, the mRNA level of *IL1B*, *TNF*, and *IFNG* was considerably decreased by the addition of 1,25(OH)_2_D_3_ (*P* < 0.05), whereas the gene expression of *IL10* was significantly (*P* < 0.05) increased by the addition of 1,25(OH)_2_D_3_ (**Figure 2C**). The treatments of 1,25(OH)_2_D_3_ significantly (*P* < 0.05) suppressed the expression of ASC, phos-IκB ^ser 32 ser36^/IκB, and intranuclear NF-κB p65 (**Figure 2G, H**).

#### 3.2.3 Apoptosis-related parameters *in vitro*

The TUNEL assay was conducted to test the apoptosis level of primary intestinal epithelial cells. DAPI stained both apoptotic and non-apoptotic cells blue, and only apoptotic nuclei had red fluorescence localized by TMR-5-dUTP incorporation. The TUNEL assay of cell climbing slides is shown in **Figure 2E**. Under the excitation of ultraviolet light, the nuclei stained by DAPI are blue, and the positive apoptotic nuclei are red. 1,25(OH)_2_D_3_ obviously reduced the fluorescence intensity of positive apoptotic nuclei.

With the increasing addition of 1,25(OH)_2_D_3_ in the medium, the gene expression of *CASP3* and *BAX* was significantly (*P* < 0.05) decreased, while the *BCL2* expression was remarkably (*P* < 0.05) enhanced (**Figure 2D**).

The treatment of 1,25(OH)_2_D_3_ prominently (*P* < 0.05) reduced the protein expression of CASP3 but significantly (*P* < 0.05) raised the protein expression of BCL2 (**Figure 2G, I**).

#### 3.2.4 Autophagy-related parameters *in vitro*

As shown in **Figure 2D**, the highest gene expression of *ATG16L1* was observed in treatment with 1 nM 1,25(OH)_2_D_3_ (*P* < 0.05). The treatments of 1,25(OH)_2_D_3_ significantly (*P* < 0.05) enhanced the protein expression of MAP1LC3B and ATG16L1 (**Figure 2I**).

Immunofluorescence was conducted to analyze the intracellular localization of MAP1LC3B. Following the immunofluorescence results of cell climbing slides (DAPI, blue; MAP1LC3B fluorescence, red), the additions of 1,25(OH)_2_D_3_ in the cell promoted the fluorescence of MAP1LC3B (**Figure 2F, G**).

### 3.3 RNA interference of VDR *in vivo*

#### 3.3.1 Efficiencies of VDR siRNAs *in vivo*

**Figure 3:**
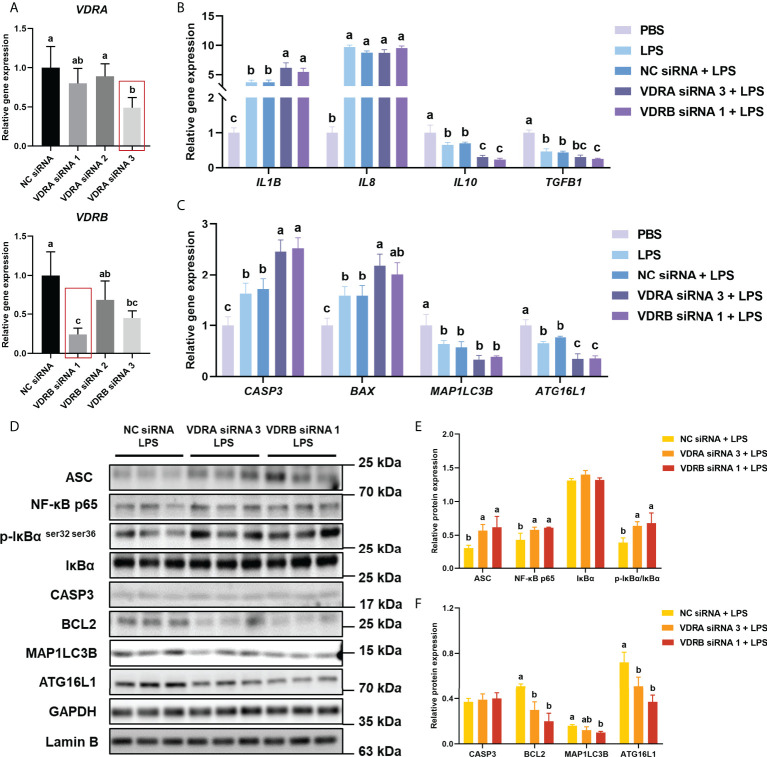
VDR knockdown *in vivo* on inflammation, apoptosis, and autophagy in the intestine. **(A)** The relative mRNA expression level of VDRA&B in the intestine treated with VDRA&B siRNAs. **(B)** The mRNA expression levels of *IL1B*, *IL8*, *TGFB1*, and *IL10*. **(C)** The mRNA expression levels of *CASP3*, *BCL2*, *BAX*, *MAP1LC3B*, and *ATG16L1*. **(D-F)** The level of ASC, intranuclear NF-κB p65, total IκB and phosphorylated IκB, CASP3, BCL2, MAP1LC3B, and ATG16L1 were analyzed and quantitated by western blot. The blots of ASC, IκBα, p-IκBα ^ser32 ser36^, CASP3, BCL2, MAP1LC3B, and ATG16L1 were used for GAPDH loading control, while the blot of NF-κB p65 was used for Lamin B loading control. Error bars of columns denote SD (n = 3), and columns with different letters above them are significantly different (*P* < 0.05).

As shown in **Figure 3A**, the result indicated that the VDRA siRNA 3 and VDRB siRNA1 were the most efficient (*P* < 0.05) duplex for knocking down VDRA&B expression in the intestine of turbot (about 50% and 70%, respectively), which were selected for the following experiments.

#### 3.3.2 Inflammation-related parameters after VDR RNAi *in vivo*

Compared with the PBS group, the LPS injection significantly (*P* < 0.05) enhanced the gene expression of pro-inflammatory cytokines (*IL1B* and *IL8*) but prominently (*P* < 0.05) suppressed the mRNA level of anti-inflammatory cytokines (*IL10* and *TGFB1*) in the intestine of turbot. Compared with NC siRNA+LPS group, the gene expression of *IL1B* significantly (*P* < 0.05) increased, and *IL10* and *TGFB1* significantly (*P* < 0.05) decreased in the siVDR treated groups (**Figure 3B**). The results of western blot showed that the knockdown of VDRA&B remarkably (*P* < 0.05) activated the NF-κB signalling in terms of a significant (*P* < 0.05) increase of ASC, intranuclear NF-κB p65, and phos-IκB ^ser 32 ser36^/IκB (**Figure 3D, E**).

#### 3.3.3 Apoptosis-related parameters after VDR RNAi *in vivo*

The gene expression of *CASP3* and *BAX* after LPS stimulation was significantly higher (*P* < 0.05) than the expression in the PBS group. The VDRA&B knockdown significantly (*P* < 0.05) induced the gene expression of *CASP3* and *BAX* compared to the NC siRNA when injected by LPS (**Figure 3C**). A similar result was also observed in western blot analysis that the protein expression of BCL2 was significantly (*P* < 0.05) lower in the VDRA siRNA 3+LPS and VDRB siRNA 1+LPS than that in the NC siRNA+LPS group (**Figure 3D, F**).

#### 3.3.4 Autophagy-related parameters after VDR RNAi *in vivo*

The injection of LPS prominently (*P* < 0.05) reduced the gene expression of *MAP1LC3B* and *ATG16L1* in the intestine of the turbot. Compared with the NC siRNA+LPS group, the knockdown of VDRA&B led to a significant (*P* < 0.05) reduction of gene and protein expression of MAP1LC3B and ATG16L1(**Figures 3C, D, F**).

## 4 Discussion

According to the previous diagnostic criteria of enteritis in Atlantic salmon (*Salmo salar* L.) (37) and turbot (38), typical intestinal enteritis histomorphology was observed in the VD_3_ deficiency group in terms of shortened mucosal fold, disordered goblet cells, and widened lamina propria. Besides, a reduced expression of tight junction proteins, decreased mucin secretion, overexpression of pro-inflammatory cytokines, and suppressed immune response were also involved in the pathological process of enteritis in fish (3, 4, 6, 39). In the present study, suppressed secretion of intestinal mucus and lower expression of tight junction protein led to the intestinal mucosal barrier dysfunction in turbot, which was also observed *in vitro* (reduction in the gene expression of tight junction proteins). Moreover, the mRNA levels of pro-inflammatory and anti-inflammatory cytokines in VD_3_ deficiency treatment were significantly enhanced or suppressed *in vivo* and *in vitro*, respectively. In addition, the *in vivo* results showed that VD_3_ deficiency contributed to the reduced intestinal immune-related enzyme activities of ACP, ALP, and LYZ activities. These results showed that the deficiency of VD_3_ in diet could induce intestinal enteritis of turbot, which was consistent with the previous studies that diets with sufficient VD_3_ are the basis for maintaining intestinal immune function, and VD_3_ deficiency provoked an intestinal inflammatory response in European sea bass (*Dicentrarchus labrax* L.) and Chinese mitten crabs (5, 8).

In the present study, VD_3_ deficiency in diet activated the NF-κB signalling pathway of turbot by blocking NF-κB nuclear translocation and reducing IκBα phosphorylation. Generally, activation of NF-κB signalling activates the inflammasome by promoting the expression of the inflammasome adaptor ASC, which may also lead to increased expression and secretion of interleukins (40). In the present study, dietary administration of VD_3_ suppressed intestinal inflammation by down-regulating the NF-κB/inflammasome signalling. In male Sprague–Dawley rats, the treatment of VD_3_ suppressed the exercise-induced muscle inflammation through the modulation of MAPK and NF-κB involved with VDR (41). The study in abalone (*Haliotis discus hannai*) also showed that VD_3_ could inhibit inflammation by significantly decreasing the phosphorylation of IKK and IκB and further blocking nuclear translocation of NF-κB (32).

A steady accumulation of studies showed that apoptosis and autophagy are often regulated by similar pathways and usually cooperated in a balanced interplay or facilitated cellular destruction in a complementary fashion (42). Effects of VD_3_ on apoptosis are diverse in different studies because of the different experimental procedures, concentrations of VD_3,_ and the species of animals. On the one hand, VD_3_ could increase apoptosis in the treatment of cancer, obesity, and inflammatory bowel disease (43–45). On the other hand, studies in the murine showed that the exogenous VD_3_ attenuated the cell apoptosis in LPS-induced lung injury and hippocampal apoptosis induced by kainic acid and pentylenetetrazol (46, 47). DNA fragmentation assay is an apparent indicator of apoptosis, and one of the most distinctive characteristics is the 180 to 200 bp fragments (35). Under the inflamed circumstance in the intestine, diets with VD_3_ supplementation prominently decreased the relative density of the DNA ladder, especially the 180 to 200 bp DNA band, indicating that dietary VD_3_ could reduce the intestinal apoptosis of turbot in this study. Similarly, the TUNEL assay showed that VD_3_ reduced the fluorescence intensity of positive apoptotic nuclei, indicating that the administration of VD_3_ in turbot was crucial to ameliorate DNA damage in apoptosis. In the process of apoptosis activation, BCL2 blocked the release of cytochrome C by preventing the pores in the mitochondrial outer membrane formed by the combo of BAX and BCL2 Antagonist/Killer (BAK) proteins, which inhibited the activation of the caspases for dismantling the cell (48–52). In this study, VD_3_ deficiency resulted in the higher expression of CASP3 and BAX, and the lower expression of BCL2. Similar results were also observed in the study on MCF-7 breast cancer cell line that the regulation of VD_3_ on apoptosis was BAX and BCL2 depended (53). The present results indicated that intestinal apoptosis could be enhanced in response to the aggressive intestinal inflammation induced by VD_3_ deficiency, and this process relied on activating BAX/BAK combo and inhibiting BCL2.

Autophagy within the epithelium controlled inflammation-induced apoptosis and barrier integrity to limit chronic intestinal inflammation (54). ATG16L1 contributed to the addition of lipid moieties to the ubiquitin-like molecule MAP1LC3B, which promoted autophagosome formation and function (55). The present results showed that VD_3_ deficiency suppressed the expression of ATG16L1 and MAP1LC3B, and confocal images of MAP1LC3B fluorescence *in vitro* showed that the fluorescence reaction was significantly reduced without VD_3_ stimulation. And the reduction of intestinal autophagy status could be alleviated by the addition of VD_3_. Taken these results together, VD_3_ can suppress the activation of apoptosis but enhance autophagy in turbot. Moreover, the mutually exclusive cellular states of apoptosis and autophagy were also observed in abalone, in which dietary VD_3_ can significantly increase the expression of autophagy-related proteins but decrease the expression of apoptosis-related proteins (32).

Previous studies in mammals demonstrated that the VDR was involved in the inhibition of NF-κB nuclear translocation, which suppressed the expression of pro-inflammatory cytokines (56, 57). VDR also plays an important role in apoptosis. For example, the VDR/ERK signalling pathway inhibited the apoptotic cascade in hippocampal CA1 neurons of global cerebral ischemia rats (58). VDR can also inhibit high glucose-induced endothelial cell apoptosis by inhibiting oxidative stress (59). In addition, VDR may play a crucial role in vitamin D regulation of autophagy in hepatitis C virus viral infection (60). The current results showed that the gene expression of VDR was up-regulated by dietary VD_3_, and the knockdown of VDR in turbot aggravated the LPS-induced intestinal inflammatory response and apoptosis, while the reduced autophagy led by the VDR silencing was further declined. Thus, VD_3_/VDR is involved in alleviating the intestinal inflammatory response of turbot and maintaining the dynamic balance of apoptosis and autophagy.

## 5 Conclusion

The present study showed that VD_3_ deficiency in diet could induce intestinal enteritis of turbot. The inflammatory response caused by VD_3_ deficiency was regulated by the NF-κB/inflammasome pathway; interestingly, the regulation of VD_3_ on intestinal inflammatory response may be related to intestinal epithelial cell apoptosis and autophagy, establishing an antagonism of apoptosis and autophagy. Further, the regulation of VD_3_ on the inflammatory response, apoptosis, and autophagy was VDR-depended. Besides, excessive VD_3_ (4000 IU/kg) did not adversely impact turbot as in mammals, and further research relating to VD_3_ metabolism is needed to explain these results.

## Data availability statement

The original contributions presented in the study are included in the article/**Supplementary Material**. Further inquiries can be directed to the corresponding authors.

## Ethics statement

The animal study was reviewed and approved by Institutional Animal Care and Use Committee of the Ocean University of China.

## Author contribution

Conceptualization: ZC, DH, JD, and YZ; Methodology: ME, OJ, WZ, QA, KM, JD, and YZ; Formal analysis and investigation: ZC, DH, PY, and GL; Writing - original draft preparation: ZC; Writing - review and editing: ZC, DH, ME, OJ, WZ, QA, KM, JD, and YZ; Funding acquisition: YZ; Supervision: JD and YZ. All authors contributed to the article and approved the submitted version.

## Funding

This work was supported by the National Natural Science Foundation of China (No. 31872577); the National Key R&D Program of China (2019YFD0900104); China Agriculture Research System (Grant No. CARS 47-G10). ZC appreciated the financial support from the China Scholarship Council by a State Scholarship Fund (No.201806330100).

## Conflict of interest

The authors declare that the research was conducted in the absence of any commercial or financial relationships that could be construed as a potential conflict of interest.

The reviewer SZ declared a shared affiliation with authors GL and JD to the editor at the time of review.

## Publisher’s note

All claims expressed in this article are solely those of the authors and do not necessarily represent those of their affiliated organizations, or those of the publisher, the editors and the reviewers. Any product that may be evaluated in this article, or claim that may be made by its manufacturer, is not guaranteed or endorsed by the publisher.

## Abbreviations

ACP, acid phosphatase; ALP, alkaline phosphatase; ANOVA, one-way analysis of variance; ATG16L1, Autophagy Related 16 Like 1; BAK, BCL2 Antagonist/Killer; BAX, BCL2 Associated X, Apoptosis Regulator; BCA, bicinchoninic acid; BCL2, BCL2 Apoptosis Regulator; BSA, Bovine Serum Albumin; CASP3, Caspase 3; CLDN4, Claudin 4; DSS, dextran sulfate sodium; GAPDH, Glyceraldehyde-3-phosphate dehydrogenase; H&E, hematoxylin and eosin; HBSS, Hank’s Balanced Salt Solution; IFNG, Interferon Gamma; IL10, Interleukin 10; IL1B, Interleukin 1 Beta; IL8, Interleukin 8; Junctional adhesion molecule 1; LYZ, lysozyme; MAP1LC3B, Microtubule Associated Protein 1 Light Chain 3 Beta; MUC2&18, Mucin 2&18; RNAi, RNA interference; RPSD, RNA polymerase II subunit D; SD, standard deviation; TGFB1, Transforming Growth Factor Beta 1; TNF, Tumor Necrosis Factor; TRIC, Tricellulin; JAM1, Junctional adhesion molecule 1; VD_3_, Vitamin D_3_; VDRA&B, Vitamin D Receptor A&B; ZO1, Zonula Occludens 1.
