# Supplementary figures and images for: Vitamin D3 deficiency induced intestinal inflammatory response of turbot through nuclear factor-κB/inflammasome pathway, accompanied by the mutually exclusive apoptosis and autophagy
Source: Front Immunol. 2022 Sep 8;13:986593. doi: 10.3389/fimmu.2022.986593 (PMC9493454; doi:10.3389/fimmu.2022.986593)

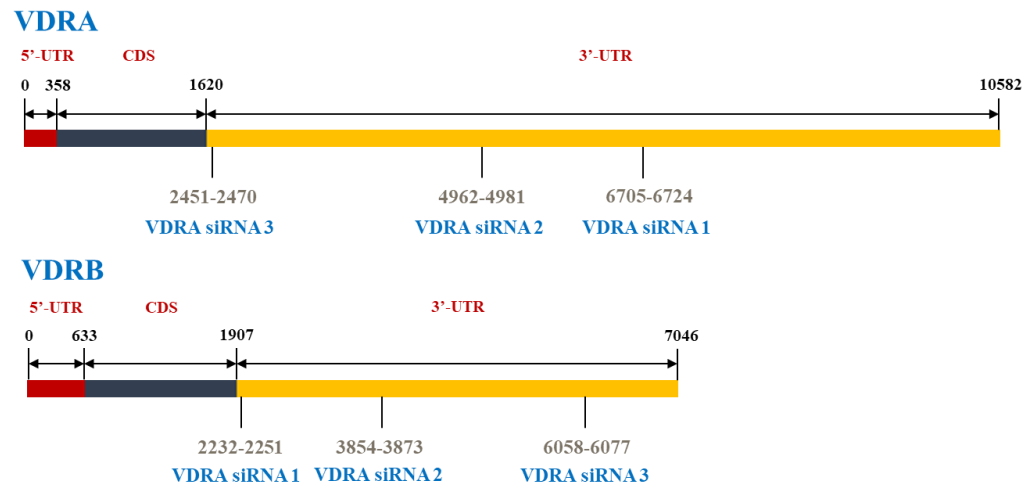

**Figure S.** Region of mRNA where the siRNAs of VDRA&B are designed

Supplement: Supplementary file 1 [file DataSheet_1.pdf]
